# 

**Published:** 1896-12

**Authors:** 


### Meeting of the South Texas Medical Association.

DEAR DOCTOR:—You are invited to be present and participate in the meeting of the South Texas Medical Association, on December 9th, 1896, at 10 a. m., in the Annex of the Capitol Hotel, Houston, Texas.

The following is a part of the program:

Meeting called at 10 a. m., by Joseph A. Mullen, M. D., Chairman of Organization Committee.

Prayer by Rev. Dr. Aves, Houston.

Address by Dr. Robert T. Morris, President Houston District Medical Society.

Address in Response, for the Medical Fraternity of Houston, by Dr. Max Urwitz.

1. "Antiseptic Treatment of Typhoid Fever," by H. A. West, M. D., Galveston.

2. "Radical Treatment of Typhoid Fever," by Frank B. King, M. D., Houston.

3. "Some Practical Points in the Treatment of Ophthalmia in the New Born," by Vard H. Hulen, M. D., Galveston.

4. "Such a Simple Thing as Cutting off a Leg," by S. C. Red, M. D., Houston.

5. "Isolation of Tubercular Patients," by Robt. McElroy, M. D., Houston.

6. "Malarial Hæmaturia," by Bat Smith, M. D., Wharton.

7. "Stricture of the Rectum," by R. W. Knox, M. D., Houston.

8. "Nose-Bleeding in Children," by Joseph A. Mullen, M. D., Houston.

9. "Experience of a Surgeon in a Railway Hospital," by Joseph R. Stuart, M. D., Houston.

10. "Placenta Praevia," by William Olive, M. D., Houston.

11. "Appendicitis," by J. P. Hendricks, M. D., Huntsville.

12. "Entero-Colitis in Children," by J. W. Scott, M. D., Houston.

COMMITTEE.

